# Graphic Illustrations of Abortion, and the Diseases of Menstruation

**Published:** 1833-10

**Authors:** 


*Graphic Illustrations of Abortion, and the Diseases of Menstruation.* By A. B. GRANVILLE, M.D. F.R.S. London, 1833. 4to.

How far a work of science is demanded by the state of knowledge, is a question which it behoves every author to ask himself, previous to offering his lucubrations to a public already possessed of an accumulation of information from former sources, and therefore more fastidious concerning the character of novelty which any present production may bear. The title of Dr. Granville's volume might very probably cause the question to be raised by all those English medical men who have seen and admired (and who has not?) the splendid illustrations of the gravid uterus, published by Dr. Hunter. Our author, anticipating the probability of such a question, has himself broached it in the "advertisement"

to the Graphic Illustrations; and, as his own answer must be that of every impartial student, we give it in his own words:

"Not to speak of the many authors on matters of this description who preceded Dr. Hunter and Professor Soemmering, in giving representations of the human embryo in many of its metamorphoses, such as Ruysch, Noortwyk, Albinus, Krapft, Wrisberg, Camper, Blumenbach, Denman, and others, I am ready to admit that both Hunter and Soemmering had given to the world delineations, such as are here referred to, which ought to satisfy the medical profession. The one, in his splendid work on the 'Gravid Uterus,' a gigantic folio edition; the other, in his no less valuable folio plates of the human embryo. But, unfortunately, neither of these works can be considered as accessible to the generality of professional readers, on account of their high price and rarity; and even if either were accessible, the subjects therein treated are viewed differently from those collected in the present work. Those works, moreover, present no coloured specimen of delineation of parts, most of which, if not all, from that circumstance lose their principal value; and, as productions of art, they are not equal to what Mr. Parry has enabled me to offer to the public."

Again, as to the want of similar plates, Dr. Granville says,

"I support the affirmative by appealing to all those of the profession who have had occasion to be consulted in cases of abortion, and the expulsion of those singular productions of the uterine cavity which seem connected with menstruation or faulty conception. Let them say whether a work in which the principal of the infinitely varied aborted human ova, and of the uterine productions alluded to, are faithfully represented, be not likely to be useful; whether, in fact, it be not wanted; or whether it exists already anywhere?"

The reader will find that, in these answers, Dr. Granville has paid all the respect that is due to his predecessors in this path of investigation, and has not discovered too much of the self-complacency which the labour of years permits an author to assume. Nor will this be the less evidently true on perusing the letter-press, and examining the plates which accompany it.

The letterpress is composed of physiological "prolegomena," contained in 102 propositions; with explanations of the pathological plates, and remarks upon them.

In the Prolegomena, the author combats the "antiquated theories" concerning the fecundation and development of the ovum; theories which, he says, "the great and rapid strides which physiology has made in France, as well as in Germany, have contributed to explode." And, when we peruse the arguments and authorities cited in this part of the work, to establish more modern doctrines, we cannot but think it essential that every medical man, reared in the belief of older ones, should learn from these propositions of Dr. Granville on how uncertain a ground his own creed is founded. In order to give such a reader, as well as the one of more modern notions, some idea of the doctrines advanced in the work, we transcribe a few of the propositions.

"The ovulum has been traced, after fecundation, into the cavity of the womb, where the external covering becomes what Boer has called 'the cortical membrane' (cortex ovi of the present work), improperly considered as a uterine production by preceding writers, and denominated the reflected caducous or deciduous membrane." (Prop. 20.)

"The more intimate covering of the yellow body of the ovulum, that which closely invests its surface and appears only after fecundation, is afterwards changed into what is called the shaggy chorion." (Prop. 21.)

"The cortical membrane is destined to be absorbed during the first months of utero-gestation, thus exposing the next membrane to the contact of the uterine lining (decidua), with which a connexion takes place in that part where the placenta is to be formed. In that part, however, the cortex ovi is never altogether obliterated, but only made thinner; and in process of time it is converted into a mere pellicular envelope, which not only serves to divide the filiform vessels of the chorion into groups, or cotyledons, in order to form the placenta, but also covers all over those cotyledons or groups of vessels." (Prop. 26.)

"On the cortex bursting, the lanuginous or fibrillous membrane within it (see prop. 21,) is exposed, when the fibrils will forthwith entwine themselves with the flocculi of the decidua, and thus the ovulum fastens itself to the uterus by one or more contiguous points. Carus." (Prop. 43.)

"The membrane having these fibrils on its surface has been called the chorion; and from the circumstance that these fibrils, both before the cortex which lies over them has burst, as well as afterwards, serve to promote the nourishment of the fœtus, I have styled it the nutritive membrane, or involucrum of the fœtus." (Prop. 44.)

"The amnion is a sac formed by the reflected epidermis of the embryo, (Velpeau, Boer, Pockels.) It does not exist before the twelfth day (Velpeau.) At the eighteenth day it is found as a bladder placed on the back of the embryo, and continuous to it along its edges or sides, and at its extremities (Velpeau.) It has been distinctly seen on the twelfth day (Pockels.) It is then not a concentric membrane with the chorion, but a vesicle, on the outside of which the embryo rests as on a bed. Until the day in question, the embryo is connected to the vesicular amnion at the back, by a cellular transparent membrane. From that time till the sixteenth day, the embryo progressively gets into the cavity of the amnion, which before was connected with the chorion by one of its pyriform extremities, while the other conical extremity penetrates slowly into the albuminous fluid of the chorion (Pockels.)" (Prop. 54.)

"The nervous system is not developed, beginning at the centre, and proceeding towards the circumference of the embryo, but the reverse. Thus the lateral nerves of the head, trunk, and pelvis, are already formed, when the cerebro-spinal system is in a liquid state. It follows hence, that those nerves cannot be considered (as it has all along been supposed,) in the light of emanations from, but as distinct bodies proceeding to, that particular portion of the nervous system (Serres.") (Prop. 69.)

Though thus abounding in modern authorities, it must not be supposed that Dr. Granville has omitted to verify by his own observations those points which have been recently advanced: on the contrary, his personal experiments continually come to bear upon such, as well as to elucidate doctrines purely his own. The latter is more particularly seen in the propositions included within the 77th and 88th. In these the formation and structure of the placenta are well laid down, and his own experiments detailed in a clear and forcible manner.

Suffice it to state, in the author's own words, (Prop. 89,) that his "view of the real structure of the placenta differs from that of Dr. Hunter only in the non-adoption of that great man's notion that continuous vessels go from the uterus, *through* the decidua, direct into appropriate cells, or laminæ, where he supposed that the arteries deposited their blood, which the veins pumped back into the uterine vessels' system of the mother, after it had served the purpose of bathing the terminal or cotyledonic vessels of the fœtus."

With regard to the fœtus, the author holds the opinion that it is independent of the mother *quoad* life, but dependent *quoad* nutrition. (Prop. 100.)

Respecting the plates, we can only add our tribute of praise to that which has already been bestowed upon them.

